# Transcriptomic analysis of *Lycium ruthenicum* Murr. during fruit ripening provides insight into structural and regulatory genes in the anthocyanin biosynthetic pathway

**DOI:** 10.1371/journal.pone.0208627

**Published:** 2018-12-07

**Authors:** Yan-Jun Ma, Hui-Rong Duan, Feng Zhang, Yi Li, Hong-Shan Yang, Fu-Ping Tian, Xue-Hui Zhou, Chun-Mei Wang, Rui Ma

**Affiliations:** 1 College of Forestry, Gansu Agricultural University, Lanzhou, China; 2 College of Agronomy, Gansu Agricultural University, Lanzhou, China; 3 Lanzhou Institute of Husbandry and Pharmaceutical Science, Chinese Academy of Agricultural Sciences, Lanzhou, China; ICAR-Indian Institute of Agricultural Biotechnology, INDIA

## Abstract

Fruit development in *Lycium ruthenicum* Murr. involves a succession of physiological and biochemical changes reflecting the transcriptional modulation of thousands of genes. Although recent studies have investigated the dynamic transcriptomic responses during fruit ripening in *L*. *ruthenicum*, most have been limited in scope, and thus systematic data representing the structural genes and transcription factors involved in anthocyanin biosynthesis are lacking. In this study, the transcriptomes of three ripening stages associated with anthocyanin accumulation, including S1 (green ripeness stage), S2 (skin color change) and S3 (complete ripeness stage) in *L*. *ruthenicum* were investigated using Illumina sequencing. Of a total of 43,573 assembled unigenes, 12,734 were differentially expressed during fruit ripening in *L*. *ruthenicum*. Twenty-five significantly differentially expressed structural genes (including *PAL*, *C4H*, *4CL*, *CHS*, *CHI*, *F3H*, *F3’H*, *F3’5’H*, *DFR*, *ANS* and *UFGT*) were identified that might be associated with anthocyanin biosynthesis. Additionally, several transcription factors, including MYB, bHLH, WD40, NAC, WRKY, bZIP and MADS, were correlated with the structural genes, implying their important interaction with anthocyanin biosynthesis-related genes. Our findings provide insight into anthocyanin biosynthesis and regulation patterns in *L*. *ruthenicum* and offer a systematic basis for elucidating the molecular mechanisms governing anthocyanin biosynthesis in *L*. *ruthenicum*.

## Introduction

As the largest subclass of water-soluble pigments, anthocyanins determine the coloration of flowers and fruits [1, 2]. Anthocyanins are synthesized via the flavonoid pathway and possess a multitude of biological roles, such as protecting plants against solar exposure and ultraviolet radiation, and also possess antioxidative capacity and free radical scavenging activity [3, 4]. Anthocyanins also influence dietary preferences in humans, as they have potential health properties associated with the prevention of cancer and cardiovascular diseases [5].

The anthocyanin biosynthetic pathway is catalyzed stepwise by 11 major types of structural genes, including *PAL* (phenylalanine ammonia-lyase), *C4H* (cinnamate-4-hydroxylase), *4CL* (4-coumaroyl: CoA-ligase), *CHS* (chalcone synthase), *CHI* (chalcone isomerase), *F3H* (flavanone 3-hydroxylase), *F3'H* (flavonoid 3'-hydroxylase), *F3'5'H* (flavonoid 3'5'-hydroxylase), *DFR* (dihydroflavonol 4-reductase), *ANS* (anthocyanidin synthase), and *UFGT* (UDP-glucose: flavonoid 3-O-glucosyltransferase) [6–8]. The pathway has been well studied and many structural genes have been identified in plants, including in *Arabidopsis thaliana* [9], apple [10], eggplant [11], *Senecio cruentus* [12], and sweet cherry [13].

Anthocyanin structural genes are regulated by transcription factors (TFs) from the myeloblastosis (MYB), basic helix-loop-helix (bHLH), and WD40 families [14–17]. The TFs form a MYB-bHLH-WDR (MBW) complex that binds to the promoters to control structural gene transcription [18, 19]. Zinc metallochaperone-1 (ZmC1) was the first MYB TF reported in plants and plays a major role in maize anthocyanin biosynthesis [20]. The R2R3-MYB genes are thought to be key in determining the spatial and temporal patterning of anthocyanins in plants [21]. The bHLH TFs belong to the largest TF family, second to MYB TFs. A total of 162 and 167 bHLH TFs have been confirmed in *A*. *thaliana* and *Oryza sativa*, respectively [22, 23]. Plant genomes typically encode more than 200 putative WD40 proteins which are involved in fundamental mechanisms such as signal transduction, chromatin modification and transcriptional regulation [24]. Gβ and TRANSPARENT TESTA GLABRA1 (TTG1) from WD40 family are main members in plant immunity [24].

During fruit formation and ripening in many plants, anthocyanin synthesis is enhanced by the co-regulation of a suite of TFs. Anthocyanins are considered as effective markers for monitoring ripening stages and the organoleptic quality of fruits [25]. Fang et al. [8] demonstrated that the color of ‘Furongli’ plum (*Prunus salicina*) changed from green to red during ripening, which was associated with an evident increase in the anthocyanin content, being 53.85-fold higher at 135 days after flowering (DAF) in comparison to that at 105 DAF. Similar phenomena have been documented in apple (*Malus domestica*) [26], grapevine (*Vitis vinifera*) [27], strawberry (*Fragaria* spp.) [28], eggplant (*Solanum melongena*) [29] and lychee (*Litchi chinensis*) [30].

*Lycium ruthenicum* Murr. (Solanaceae), commonly known as black goji, is a perennial xero-halophyte shrub with excellent adaptability to adverse arid environments, and is widely distributed in northwestern China, Europe and Central Asia [31, 32]. This species is a good source of natural antioxidants and shows promising activity towards the prevention of tumors, hypolipidemia, and cardiovascular diseases [33, 34]. Furthermore, *L*. *ruthenicum* is considered as a superior wild source of anthocyanins, and has thus been used as a classic model for investigating anthocyanin biosynthesis and regulation, particularly with regards to fruit pigmentation [35]. The anthocyanin content in *L*. *ruthenicum* fruit was approximately 500 mg/100 g FW [33], which was much higher than some other anthocyanin-rich fruits, such as blackberry (104 mg/100 g FW) and the Cornelian cherry cultivar (223 mg/100 g FW) [28]. Several anthocyanin biosynthesis-related genes have been evaluated in *L*. *ruthenicum*. For instance, two structural genes involved in anthocyanin biosynthesis, namely *LrLAR* and *LrANR*, were isolated in *L*. *ruthenicum*, and their expressions also exhibited evident increase from the unripe stage to the color stage, implying the potential roles in anthocyanidin biosynthesis [36]. Zeng et al. [37] also identified several genes involved in anthocyanin biosynthesis in the fruits of *L*. *ruthenicum* and *L*. *barbarum* by comparative analysis. Furthermore, 83 MYB family TFs were annotated in *L*. *ruthenicum* based on an RNA-sequencing (RNA-Seq) analysis [38]. However, reports concerning the regulation of anthocyanin synthesis and the suite of structural and regulatory genes in the anthocyanin biosynthetic pathway in *L*. *ruthenicum* are limited.

In response to this limitation, the current study evaluated anthocyanin biosynthesis at three different development or ripeness stages in the fruits of *L*. *ruthenicum* using RNA-Seq. The RNA-Seq data allowed us to investigate the structural genes and TFs related to anthocyanin biosynthesis throughout fruit coloration. Our findings offer insight into the molecular mechanisms of anthocyanin biosynthesis in *L*. *ruthenicum* and provide a foundation for the future genetic engineering of improved anthocyanin content in plants.

## Materials and methods

### Plant materials

*Lycium ruthenicum* plants were collected from Shizuishan in Ningxia, China (38°56.799′ N, 106°24.711′ E, 1088 H) in March 2016 and transplanted into a horticultural field of Gansu Agriculture University, Gansu, China. All the shrubs were of similar age and were cultivated on homogenous loessal soil under the same management practices (soil management, irrigation, fertilization, pruning, and disease control). No specific permission was required for the location of Shizuishan. This study did not involve endangered or protected species.

Fruit samples were harvested at three developmental stages associated with anthocyanin accumulation, namely S1 (green ripeness stage), S2 (skin color change) and S3 (complete ripeness stage). On July 26^th^ 2017, the fruits in S1 were harvested, and the fruits in S2 and S3 were harvested after 15 days and 35 days, respectively. Three shrubs at each development stage were constituted the biological replications of the material. Five representative fruits were sampled from each shrub at the same time of the day (9–10 AM). The combined samples of fifteen berries from three shrubs were sliced and then immediate frozen in liquid nitrogen and stored at −80°C until use.

### Evaluation of the color coordinates L*, a*, and b*

To quantify the fruits color perception from different developmental stages by using CIELab color space, individual fruit was evaluated through Photoshop CS3 10.0 software, in which 5 different points were randomly selected, respectively. Three independent variables were used to quantify color comparisons. Coordinate L* represented lightness, meanwhile, coordinate a* and b* represented chromaticity (a*, green–red axis, b*, blue-yellow axis).

### Determination of anthocyanin content

The same powdered freeze-dry samples with a pestle and mortar used for the RNA extraction were used for the anthocyanin determination. Anthocyanin content was determined by the modified extinction coefficient method [27]. Briefly, approximately 0.1 g sample was ground to fine powder and extracted in a 4 mL extraction solution (1% HCl in ethanol) with oscillation at 60°C, 120 r/min for 100 min. Following centrifugation (10,000 g, 25°C) for 10 min, the supernatant was transferred into a clean tube and diluted to 25 mL with extraction solution. The absorbance of the supernatant was measured at 528 nm using a spectrophotometer (TU-1901; Beijing Puxi, Co., Ltd) [39]. Anthocyanin content was calculated as milligrams of cyanidin-3-O-glucoside equivalent per gram dry weight. The following formula was used to determine anthocyanin content: MF = [(*A* × *v* × *M*_*0*_)/(*e* × *M*_*1*_)] × 100, MF: the anthocyanin content (mg/100 g DW), *A*: absorbance, *v*: 25× dilution ratio (mL), *e*: molar extinction coefficient of cyanidin-3-O-glucoside, 26,900 [40, 41]; *M*_*0*_: molecular weight of cyanidin-3-O-glucoside, 449 [40, 41]; *M*_*1*_: dry weight (g) of the sample. The anthocyanin contents at different ripeness stages were analyzed statistically by one-way analysis of variance (ANOVA) followed by Duncan’s post-hoc tests at significance level of *P* ≤ 0.05. Statistical analyses were done using SPSS version 16.0 (IBM Corporation, USA, Chicago, 2007).

### RNA preparation

Total RNA was extracted from nine samples (three biological replicates in each treatment) using a mirVana miRNA Isolation Kit (Thermo Fisher Scientific, Waltham, MA, USA). The RNA quantity and quality were determined using a NanoDrop 2000 instrument (Thermo Fisher Scientific), and RNA integrity was evaluated using an Agilent 2100 Bioanalyzer (Agilent Technologies, Santa Clara, CA, USA), with A260/A280 ratios for all samples being greater than 2.0.

### Illumina sequencing

The three triplicate biological samples at the three stages yielded nine non directional cDNA libraries, which were prepared from 4 μg of total RNA using the TruSeq Stranded mRNA LT Sample Prep Kit (Illumina, San Diego, CA, USA). The samples with an RNA Integrity Number (RIN) ≥ 8 were selected for subsequent analysis [42]. The size and purity of the library were determined using an Agilent 2100 Bioanalyzer. These libraries were then sequenced on the Illumina HiSeq XTen sequencing platform at Shanghai Oe Biotech Co., Ltd. (Shanghai, China) and 150-bp paired-end reads were generated.

### *De novo* assembly and functional annotation

Considering the effects of error rates on data, quality control of the raw reads in FASTQ format was performed using NGS QC Toolkit software freely available at http://www.nipgr.res.in/ngsqctoolkit.html [43], firstly filtering the reads with more than 30% low-quality bases (Q-value < 20), then discarding the reads with low-quality bases (Q-value < 20) from 3’ end, lastly discarding the reads with “N” bases from 5’ end, and the reads with the length less than 50 bp were removed. Pollution test of the clean reads was fulfilled by comparing 250 thousand pairs reads to the NCBI non-redundant (Nr) database. *De novo* assembly of the high-quality reads to transcripts was performed using Trinity (vesion: trinityrnaseq_r20131110) in paired-end method. Clustering and de-redundancy of transcripts were operated by TGICL, and the longest transcript that could not be extended on either end was defined as a unigene based on the similarity and length of a sequence for subsequent analysis. The sequences were annotated using a Blastx search (*E*-value <10^−5^) from several protein databases, includingNr and nucleotide (Nt) databases (http://www.ncbi.nlm.nih.gov), Swiss-Prot (http://www.expasy.ch/sprot), Kyoto Encyclopedia of Genes and Genomes (KEGG) (http://www.genome.jp/kegg), Clusters of Orthologous Groups (COG) (http://www.ncbi.nlm.nih.gov/COG), and Gene Ontology (GO) (http://www.geneontology.org/), based on sequence similarity. The Blast2GO program (http://www.blast2go.de) was used to obtain GO annotations for the unigenes [44].

## Differential gene expression (DEG) analysis

Nine independent cDNA libraries from three different maturation stages (S1, S2, and S3) of the *L*. *ruthenicum* fruits were constructed according to the method detailedly described by Li et al. [45]. Each library was sequenced in parallel using the Illumina HiSeq XTen sequencing platform at Shanghai Oe Biotech. After removing low quality tags, including tags with unknown nucleotide “Ns”, empty tags, and tags with only one copy number, the clean tags were mapped to our transcriptome reference database. For the analysis of gene expression, FPKM (Fragments Per Kilobase of transcript per Million mapped reads) and read counts of each unigene were calculated and normalized using bowtie2 (http://bowtie-bio.sourceforge.net/bowtie2/manual.shtml) and eXpress (http://www.rna-seqblog.com/express-a-tool-for-quantification-of-rna-seq-data/). DEGs were identified using the DESeq (http://bioconductor.org/packages/release/bioc/html/DESeq.html) with estimateSizeFactors function and nbinom Test. The significant difference was tested by NB (negative binomial distribution test). A *P*-value ≤ 0.05 in multiple tests and an absolute log_2_ fold change value ≥ 2 were used as thresholds for determining significant differences in gene expression.

### Clustering analysis

To evaluate the dynamic changes in absolute expression during fruit maturation in *L*. *ruthenicum*, we performed a hierarchical clustering analysis of the expression patterns using the Short Time-series Expression Miner (STEM) program (version 1.3.11) [46]. A unique STEM clustering algorithm was used for the hierarchical clustering analysis, and the gene expression values were standardized by log-transforming the data prior to analysis.

### Correlation analysis of structural genes and TFs

Correlation analysis of anthocyanin structural genes and TFs was performed as described by Fang et al. [8]. To obtain main putative TFs related to anthocyanin biosynthesis, we only selected unigenes with a FPKM value > 50 in at least one of the three stages during fruit ripening. Pearson correlation analysis was carried out between TFs and structural genes by using the “correlate” function in SPSS 16.0 software.

### Real-time quantitative (RT-q) PCR validation

Sixteen candidate DEGs were selected to validate the transcriptome results by RT-qPCR. Total RNA was extracted from the nine samples as described above. First-strand cDNA synthesis used 0.5 μg of total RNA and followed the manufacturer’s protocol (Vazyme, R223-01). In the second step, 2 μL of 5 × HiScript II Q RT SuperMix IIa was added and then amplification was achieved in a GeneAmp PCR System 9700 (Applied Biosystems, USA). The RT-qPCR was performed using a QuantiFast SYBR Green PCR Kit (Qiagen, Germany) and finished on the LightCycler 480 II Real-time PCR System (Roche, Swiss). The *EF1a* gene (JX427553) was used as the internal control gene. Each sample was run in triplicate. Melting curve analysis was used to validate the specific generation of the expected PCR product. The primer sequences were designed in the laboratory using the software Primer premier 5.0 and synthesized by Generay Biotech Co., Ltd. (Shanghai, China) based on the mRNA sequences obtained from the NCBI database (S1 Table). The expression levels of the mRNAs were normalized to *EF1a* and the relative expression levels of the genes were calculated using the 2^−ΔΔCt^ method [47].

### Statistical analysis

Results of anthocyanin contents are presented as means ±SE (*n* = 3) and data analysis was performed by one-way ANOVA using SPSS statistical software (Ver. 16.0, SPSS Inc., Chicago, IL, USA). Duncan’s multiple range tests were used to detect differences among means at a significance level of *P* < 0.05. Pearson correlation between the RNA-Seq data and RT-qPCR data, also between structural genes and transcription factors were calculated using the “correlate” function SPSS 16.0 software.

## Results

### Anthocyanin accumulation during ripening in *L*. *ruthenicum* fruits

**Fig 1:**
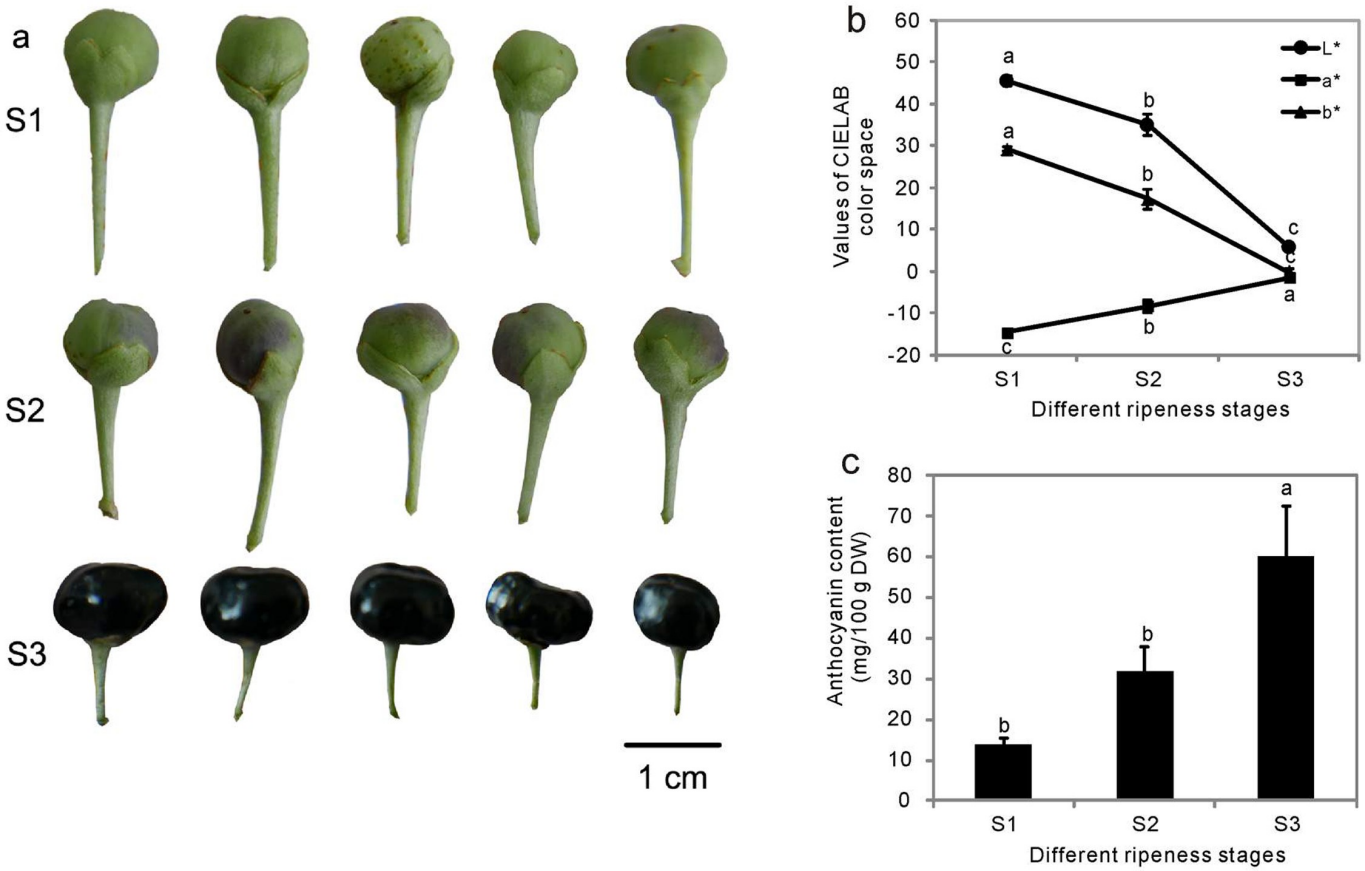
Images of *L*. *ruthenicum* fruits and anthocyanin contents in the fruits. (a) Fruits of *L*. *ruthenicum* at different ripeness stages selected for RNA-Seq. Representative photographs of fruits at the green ripeness stage (S1), skin color change (S2), and complete ripeness stage (S3). (b) Values of *L*. *ruthenicum* fruit color based on CIELab color space. L*, lightness, a*, green–red axis, b*, blue–yellow axis. (c) Anthocyanin contents in the fruits of *L*. *ruthenicum* at different ripeness stages. Values are the means ± standard error (SE) (*n* = 3). Different letters among the columns indicate significant differences (*P* ≤ 0.05).

As indicated in Fig 1A, the color of the *L*. *ruthenicum* fruits changed from green to purple-red during ripening and the flesh became pigmented. From the mean coordinates L*, a*, and b*, color differences for different developmental stages were calculated (Fig 1B). The color changes of the coordinates ranged from 45.5 to 5.8 for L*, from -14.6 to -1.6 for a*, from 29.2 to -0.2 for b*. The color coordinates L* and b* were consistently declined from S1 to S3, and conversely, the color coordinate a* increased significantly. The anthocyanin content of the *L*. *ruthenicum* fruits increased from 13.7 mg/100 g dry weight (DW) to 60.3 mg/100 g DW as ripening proceeded (Fig 1C).

### Illumina sequencing, *De novo* assembly, and functional annotation and classification

**Fig 2:**
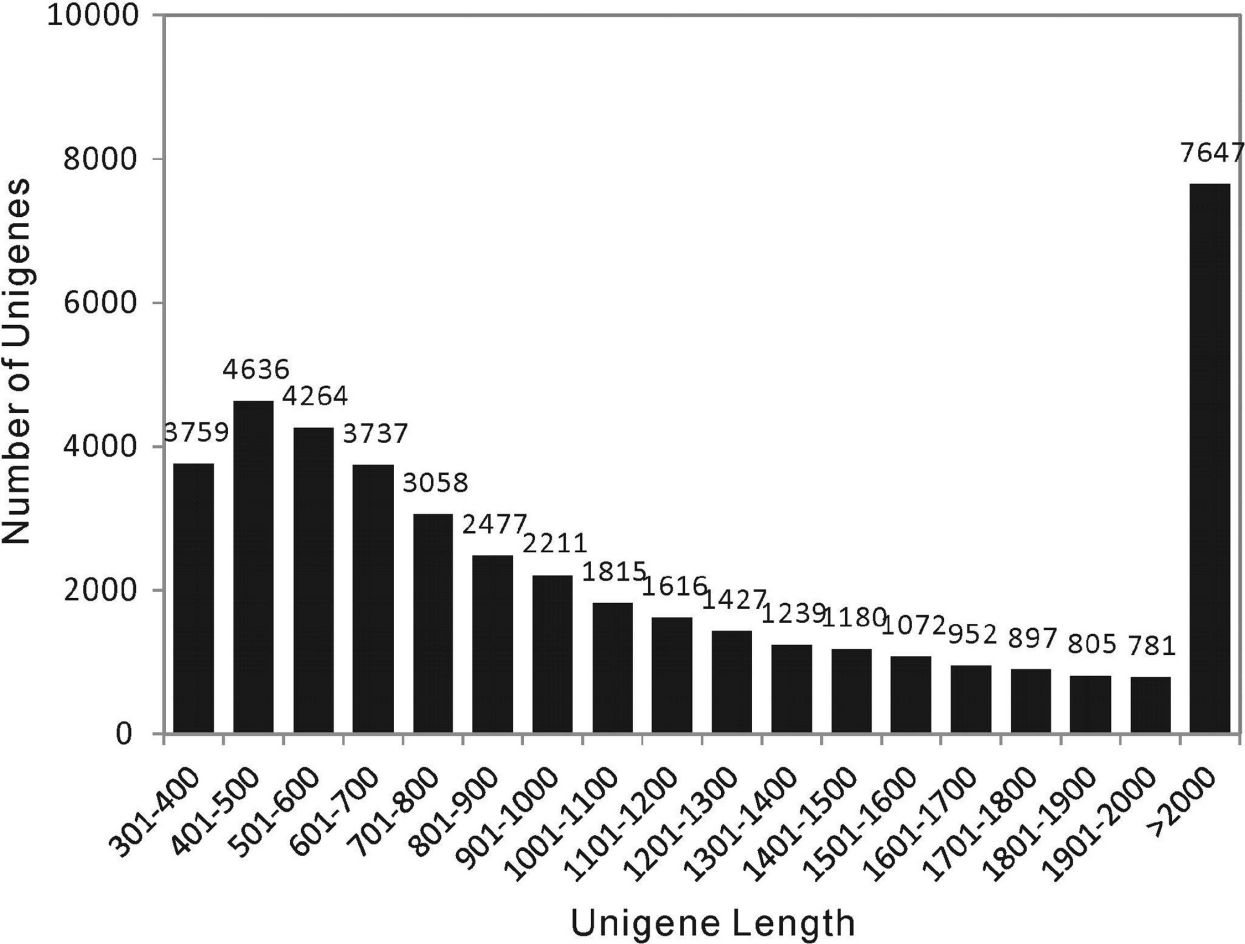
Sequence length distribution of the unigenes in the *L*. *ruthenicum* fruit transcriptomes. The *x*-axis indicates unigene length interval from 300 bp to > 2,000 bp. The *y*-axis indicates the unigenes number of each given sequence length.

To obtain complete gene expression information in the *L*. *ruthenicum* fruits, nine cDNA libraries representing the cDNA in three different stages of ripeness were subjected to RNA-Seq using an Illumina HiSeq XTen platform (Table 1). The analysis generated 50.54, 50.10, 49.60, 49.35, 49.04, 50.78, 49.02, 49.84, and 49.12 million raw 150-bp paired-end raw reads. After removing low quality reads and trimming adapter sequences, 48.96, 48.38, 48.00, 47.84, 45.83, 47.95, 45.66, 46.84, and 46.33 million clean reads were obtained from the nine libraries. Using Trinity, the clean reads were assembled into 43,573 unigenes with an average length of 1,267 bp. All of the unigenes are available in the NCBI SRA database (accession number SRR7700825). The size-distribution analysis showed that the lengths of 19,453 unigenes were greater than 1,000 bp while the lengths of 35,227 unigenes were greater than 500 bp (Fig 2). These results suggest that the quality of the unigene data was sufficient for the subsequent analyses.

**Table 1:** Summary of sequencing and *de novo* assembly.

Statistics	Samples								
	S1-1	S1-2	S1-3	S2-1	S2-2	S2-3	S3-1	S3-2	S3-3
raw reads	50537322	50097730	49603984	49354988	49037358	50782030	49019994	49839836	49124842
clean reads	48963466	48375130	47997372	47844250	45828348	47954696	45655360	46843706	46334062
Q30 percentage (%)	92.84	92.46	92.64	92.78	90.09	90.82	89.85	90.20	90.40
GC content (%)	44.00	43.00	44.00	44.00	43.00	43.50	43.50	43.00	42.00
unigenes after assembly	43573								
average length of unigenes (bp)	1263								
total nucleotides of unigenes (bp)	55017532								
N50 of unigenes (bp)	1743								

A total of 43,573 unigenes were searched against public databases, including Nr, Nt, Swiss-Prot, KEGG, COG and GO, with an *E*-value cut-off of 10^−5^. We were able to identify 54.44% of the unigenes (23,723), while the remaining unigenes (45.56%) could not be annotated with known genes (S2 Table), most likely because of an absence of genome information. Of the above 23,723 unigenes with functional annotations, 15,064, 13,128, and 4,951 unigenes could be annotated in GO, KOG, and KEGG, respectively, based on sequence homology (S2 Table). The top three matches for the *L*. *ruthenicum* fruit unigenes were with *Solanum tuberosum* (4,586, 19.47%), followed by *Capsicum annuum* (3,475, 14.75%) and *Nicotiana attenuata* (3,337, 14.16%) (S1 Fig).

### DEGs during fruit ripening

**Fig 3:**
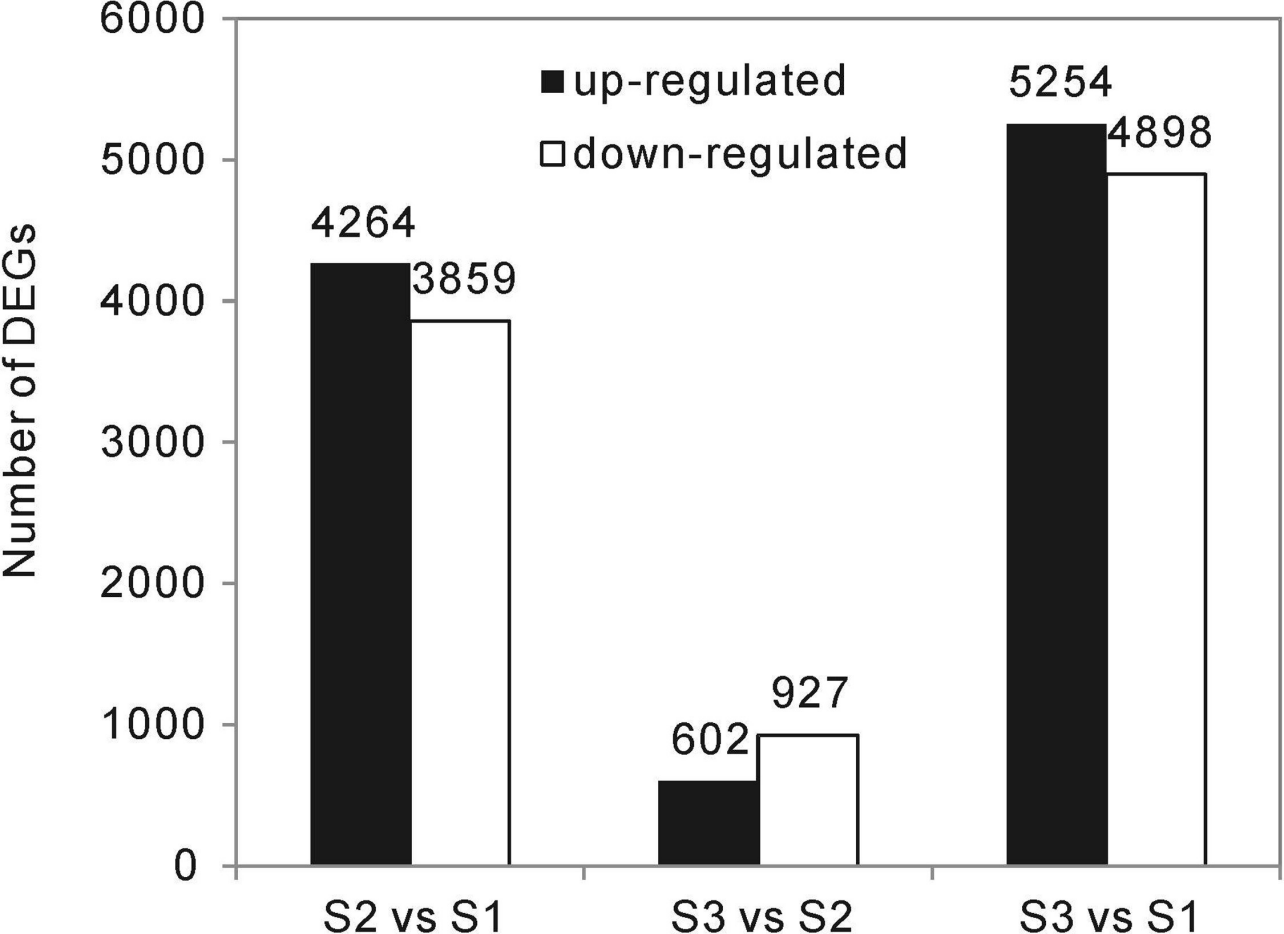
Number of DEGs between the different ripeness stages. Only transcripts exhibiting a fold change > 2 in at least one of the ripeness stage of the *L*. *ruthenicum* fruits were selected.

The unigenes from the nine libraries were mapped to the assembled transcriptome. Normalized-FPKM was used to quantify the transcript levels. The differences in gene expression were then analyzed by comparing the three different ripeness stages, using the thresholds of false discovery rate (FDR) -value <0.05 and fold change > 2, respectively (Fig 3). A total of 8,123 DEGs were identified between S1 and S2, with 4,264 up-regulated unigenes and 3,859 down-regulated unigenes, including 135 and 488 unigenes that were expressed exclusively at S1 and S2, respectively. Between S2 and S3, 1,529 DEGs were identified, with 602 up-regulated unigenes and 927 down-regulated unigenes, including 94 and 102 unigenes that were expressed exclusively at S2 and S3, respectively. Between S1 and S3, 10,152 DEGs were identified with 5,254 up-regulated unigenes and 4,898 down-regulated unigenes, including 186 and 481 unigenes that were expressed exclusively at S1 and S3, respectively. In combination, a total of 12,734 unigenes were differentially expressed during the *L*. *ruthenicum* fruit ripening process. This finding suggests that the developmental period with the most dynamic transcriptional changes was between S1 and S2, with more than half of the DEGs (63.8%) showing significant changes during this period. All of these unigene sequences can be accessed in Supporting information files: S4, S5 and S6 Tables.

### Changes in gene expression profiles during *L*. *ruthenicum* fruit maturation

**Fig 4:**
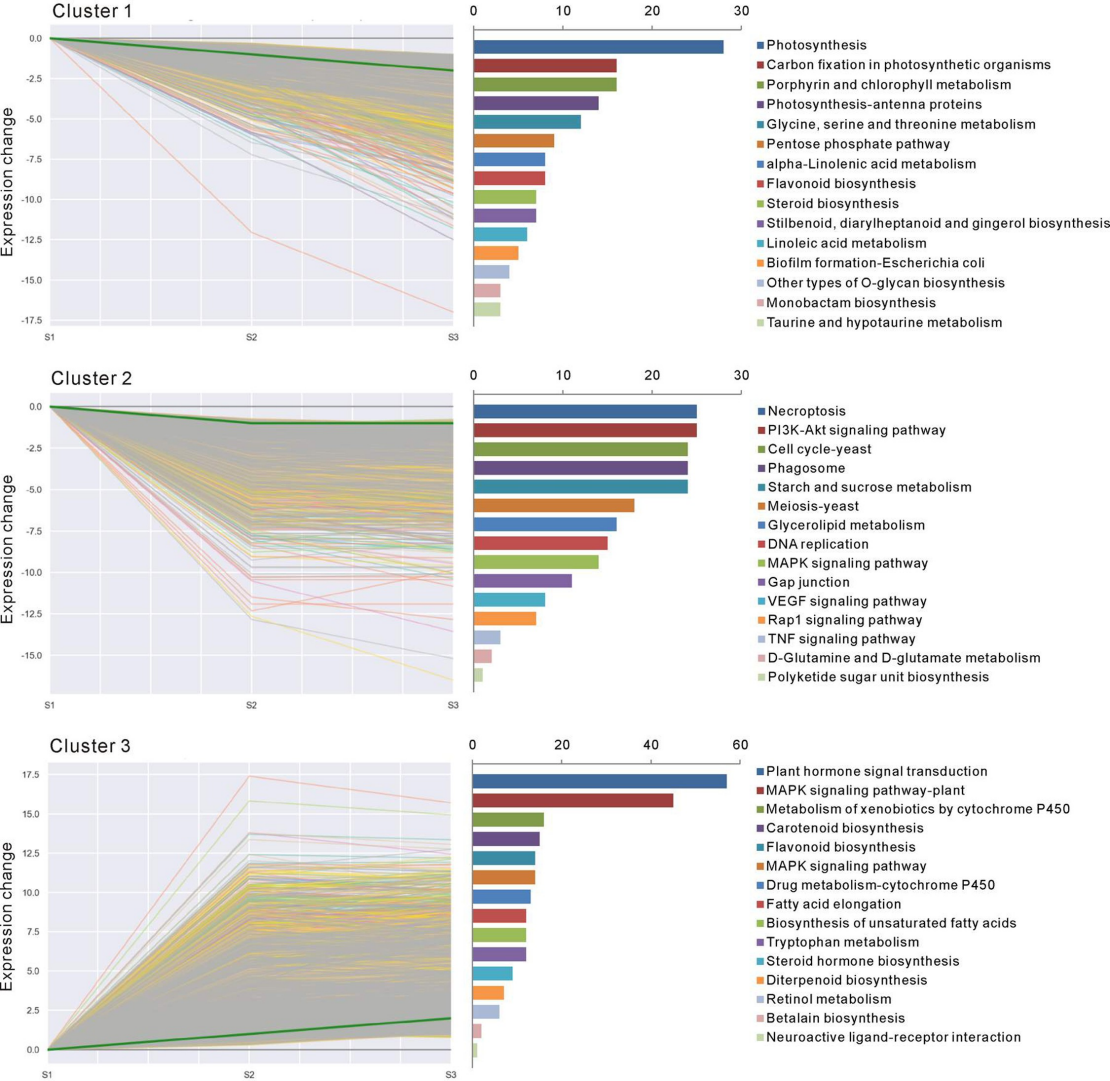
Cluster analysis of differentially expressed unigenes with significant expression profile changes at *P* ≤ 0.05 and KEGG pathway enrichment analysis. Genes coding for unknown products were not considered in the analysis. The top 15 enriched KEGG pathways are listed to the right of each cluster. The scale represents genes numbers from 15 individual KEGG pathways.

In order to gain further insights into the biological processes involved in *L*. *ruthenicum* fruit maturation, the 11,765 annotated genes commonly modulated during fruit ripening were subjected to clustering analysis, allowing for the identification of 11,703 genes exhibiting similar expression trends. Three clusters comprising 9,917 transcripts with significant differential expression at *P*-value ≤ 0.05 are illustrated in Fig 4. To provide a global description of the enriched biological pathways in each cluster of similarly regulated transcripts, we also presented an overview of the KEGG pathway enrichment (Fig 4). Cluster 1 contained 1,991 genes whose expression declined consistently throughout the ripening process, including “photosynthesis”, “carbon fixation in photosynthetic organisms”, and “porphyrin and chlorophyll metabolism.” In Cluster 2, 2,520 genes were negatively modulated from S1 to S2 and remained stable from S2 to S3, including a broad range of genes responsible for “necroptosis”, “PI3K-Akt signaling pathway”, and “cell cycle-yeast.” Cluster 3 comprised 5,406 genes whose expression increased between S1 and S2, and the top three KEGG pathways included “plant hormone signal transduction”, “MARK signaling pathway-plant”, and “metabolism of xenobiotics by cytochrome P450”, respectively. Overall, Cluster 3 constituted the largest group with 46.2% gene coverage.

### Structural genes related to anthocyanin biosynthesis

**Fig 5:**
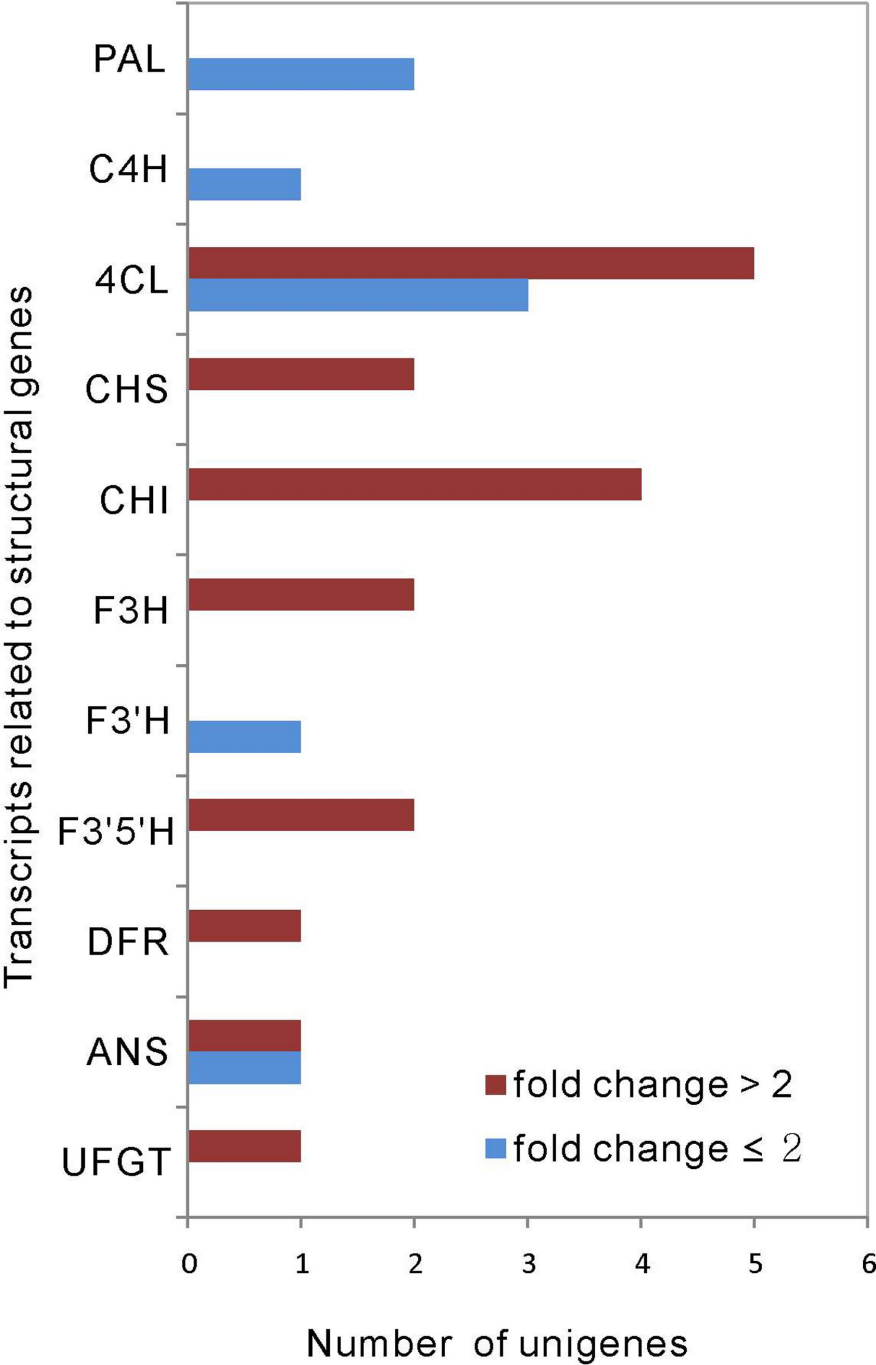
DEGs related to anthocyanin biosynthesis-related structural genes in the different fruit maturation stages of *L*. *ruthenicum*. Eleven main enzymes, including PAL, C4H, 4CL, CHS, CHI, F3H, F3'H, F3'5'H, DFR, ANS, and UFGT, were assessed. The red and blue columns indicate the DEGs with fold change > 2 and ≤ 2, respectively.

Anthocyanin biosynthesis is a dynamic and complex pathway that is regulated by a series of enzymes. In our study, 25 candidate transcripts were identified and assigned to the anthocyanin metabolic pathway (Fig 5). Eleven type main enzymes encoding structural genes, including putative *PAL*, *C4H*, *4CL*, *CHS*, *CHI*, *F3H*, *F3’H*, *F3’5’H*, *DFR*, *ANS* and *UFGT*, were further analyzed. The transcriptional levels of two *PAL* genes (CL5572Contig1 and CL15704Contig1), one *C4H* gene (CL1843Contig1), three *4CL* genes (CL9663Contig1, CL37612Contig1 and CL40022Contig1), one *F3’H* gene (CL1757Contig1), and one *ANS* gene (CL30524Contig1) exhibited similar expression patterns with fold change ≤ 2 (Table 2). Furthermore, five *4CL* genes with fold change > 2 exhibited differential expression. The CL6132Contig1, CL41122Contig1, and comp97802_c0_seq7_2 genes were down-regulated from S1 to S3. In contrast, CL32840Contig1 was upregulated from S1 to S3. The transcriptional levels of CL36675Contig1 peaked at S2. Among the genes with fold change > 2, CL25405Contig1 and CL31014Contig1 encoding *CHS*, three *CHI* genes (CL2953Contig1, CL25937Contig1, and CL29423Contig1), CL2320Contig1 and CL6713Contig1 encoding *F3H*, *F3’5’H* gene (CL6842Contig1), a *DFR* gene (CL6494Contig1), and an *ANS* gene (CL29803Contig1) displayed similar expression patterns, and were significantly upregulated during fruit maturation in *L*. *ruthenicum*. However, the expression of CL31595Contig1 encoding *CHI* and *UFGT* genes (CL11932Contig1) decreased with fruit ripening (Table 2). In total, 17 of 25 structural genes were assembled to 4 different clusters, respectively, and 11 structural genes were belonged to cluster 3 (Table 2). To analyze the anthocyanin content of the *L*. *ruthenicum* fruits with structural genes expression, we performed the correlation analysis. Among 10 different enzymes family (F3’H excepted), at least one member was highly correlated with the anthocyanin accumulation pattern, respectively (Table 2).

**Table 2:** Expression profiles, clusters distribution, and correlation analysis of the structural genes related to anthocyanin biosynthesis in *L*. *ruthenicum* fruit ripeness. “r” represents the Pearson correlation coefficient between structural genes and anthocyanin contents in different developmental fruits.

Gene name	Unigene ID	Gene length	FPKM			Cluster	r
			S1	S2	S3		
*PAL*	CL5572Contig1	2790	273.59	168.19	226.30	-	-0.328
	CL15704Contig1	321	26.95	12.02	8.08	-	-0.898
*C4H*	CL1843Contig1	3259	0.00	0.02	0.03	-	0.949
*4CL*	CL9663Contig1	330	0.10	0.00	0.40	-	0.804
	CL37612Contig1	788	4.07	6.47	3.67	-	-0.260
	CL40022Contig1	2174	329.05	188.20	246.17	-	-0.475
	CL6132Contig1	902	219.85	28.87	13.89	2	-0.832
	CL32840Contig1	2437	106.37	246.21	365.79	3	0.985
	CL36675Contig1	576	3.78	9.10	4.50	7	-0.005
	CL41122Contig1	2091	32.25	9.83	8.39	2	-0.825
	comp97802_c0_seq7_2	3393	38.68	8.23	4.60	1	-0.849
*CHS*	CL25405Contig1	1522	13.00	4121.27	6599.14	3	0.963
	CL31014Contig1	1100	3.16	601.57	809.07	3	0.920
*CHI*	CL2953Contig1	1418	28.85	219.66	241.68	3	0.847
	CL25937Contig1	1031	341.48	960.07	1424.21	3	0.978
	CL29423Contig1	1199	1.23	3.41	6.06	3	0.997
	CL31595Contig1	1242	18.88	5.86	3.24	1	-0.879
*F3H*	CL2320Contig1	1485	0.00	0.44	0.38	3	0.711
	CL6713Contig1	1555	457.17	4075.66	6282.66	3	0.964
*F3’H*	CL1757Contig1	1942	0.15	0.01	0.12	-	-0.075
*F3’5’H*	CL6842Contig1	1914	287.34	2127.58	3816.46	3	0.988
*DFR*	CL6494Contig1	1765	283.59	1368.03	2047.48	3	0.966
*ANS*	CL30524Contig1	1650	1.23	1.52	2.09	-	0.998
	CL29803Contig1	1499	30.67	1935.63	2937.17	3	0.953
*UFGT*	CL11932Contig1	506	6.85	4.11	2.90	1	-0.939

### TFs related to anthocyanin biosynthesis

TFs play crucial roles in plant growth and development. We selected TFs with FPKM ≥ 50 in at least one of the three stages during fruit ripening, from which 72 putative TFs with highly dynamic anthocyanin biosynthesis-related expressional changes were identified. To identify significant TFs that were co-expressed with the structural genes involved in anthocyanin biosynthesis, 12 structural genes with FPKM ≥ 50 were also selected. A transcription abundance correlation analysis was carried out between the 72 differentially expressed TFs and 12 structural genes from the anthocyanin biosynthetic pathway (S3 Table). Fifty-seven TFs were significantly correlated with at least one of the 12 structural genes. Sixteen TFs were evidently correlated with five or more structural genes from the anthocyanin biosynthetic pathway (Table 3), including one *MYB* gene (CL450Contig1), five *MYB*-related genes (CL25528Contig1, CL25968Contig1, CL30433Contig1, CL40520Contig1, and CL8773Contig1), two *WRKY* genes (CL11403Contig1 and CL16938Contig1), three *bZIP* genes (CL29599Contig1, CL33397Contig1 and CL39763Contig1), and five *MADS* genes (CL3655Contig1, CL6945Contig1, CL22157Contig1, CL27284Contig1 and CL30331Contig1) (S3 Table). Of these, only one of the *MYB*-related genes (CL8773Contig1) showed a negative correlation with the expression of structural genes, while most of them were positively correlated with the structural genes involved in anthocyanin biosynthesis. Interestingly, 15 of 16 TFs were assembled to cluster 3 whose expression increased between S1 and S2 (Table 3). To analyze the anthocyanin content of the *L*. *ruthenicum* fruits with the expression patterns of TFs, we performed the correlation analysis. The results showed that all the TFs were highly correlated with the anthocyanin accumulation pattern, respectively (Table 3).

**Table 3:** Expression profiles, clusters distribution, and correlation analysis of 16 TFs related to anthocyanin biosynthesis in the different fruit maturation stages of *L*. *ruthenicum*. The table shows the TFs that were significantly correlated with five or more structural genes. “r” represents the Pearson correlation coefficient between TFs and anthocyanin contents in different developmental fruits.

Gene name	Unigene ID	Gene length	FPKM			Cluster	r
			S1	S2	S3		
*MYB*	CL450Contig1	1316	0.97	173.08	274.89	3	0.962
MYB-related	CL25528Contig1	6185	38.84	69.22	89.34	3	0.970
	CL25968Contig1	1733	126.64	248.95	345.50	3	0.980
	CL30433Contig1	1585	12.37	43.96	64.96	3	0.970
	CL40520Contig1	7304	8.43	41.02	64.13	3	0.974
	CL8773Contig1	2556	78.92	48.68	24.27	1	-0.982
*WRKY*	CL11403Contig1	2870	7.51	113.12	180.32	3	0.967
	CL16938Contig1	4338	36.71	67.51	82.44	3	0.947
*bZIP*	CL29599Contig1	1551	170.15	287.47	377.57	3	0.979
	CL33397Contig1	2324	28.67	61.42	88.41	3	0.983
	CL39763Contig1	2305	111.43	217.45	296.01	3	0.977
*MADS*	CL3655Contig1	2204	10.88	88.25	124.73	3	0.945
	CL6945Contig1	3136	26.77	118.73	184.39	3	0.975
	CL22157Contig1	3379	128.49	364.03	532.25	3	0.975
	CL27284Contig1	1903	42.42	92.21	116.50	3	0.947
	CL30331Contig1	1667	1.59	377.28	612.50	3	0.966

### Experimental validation

**Fig 6:**
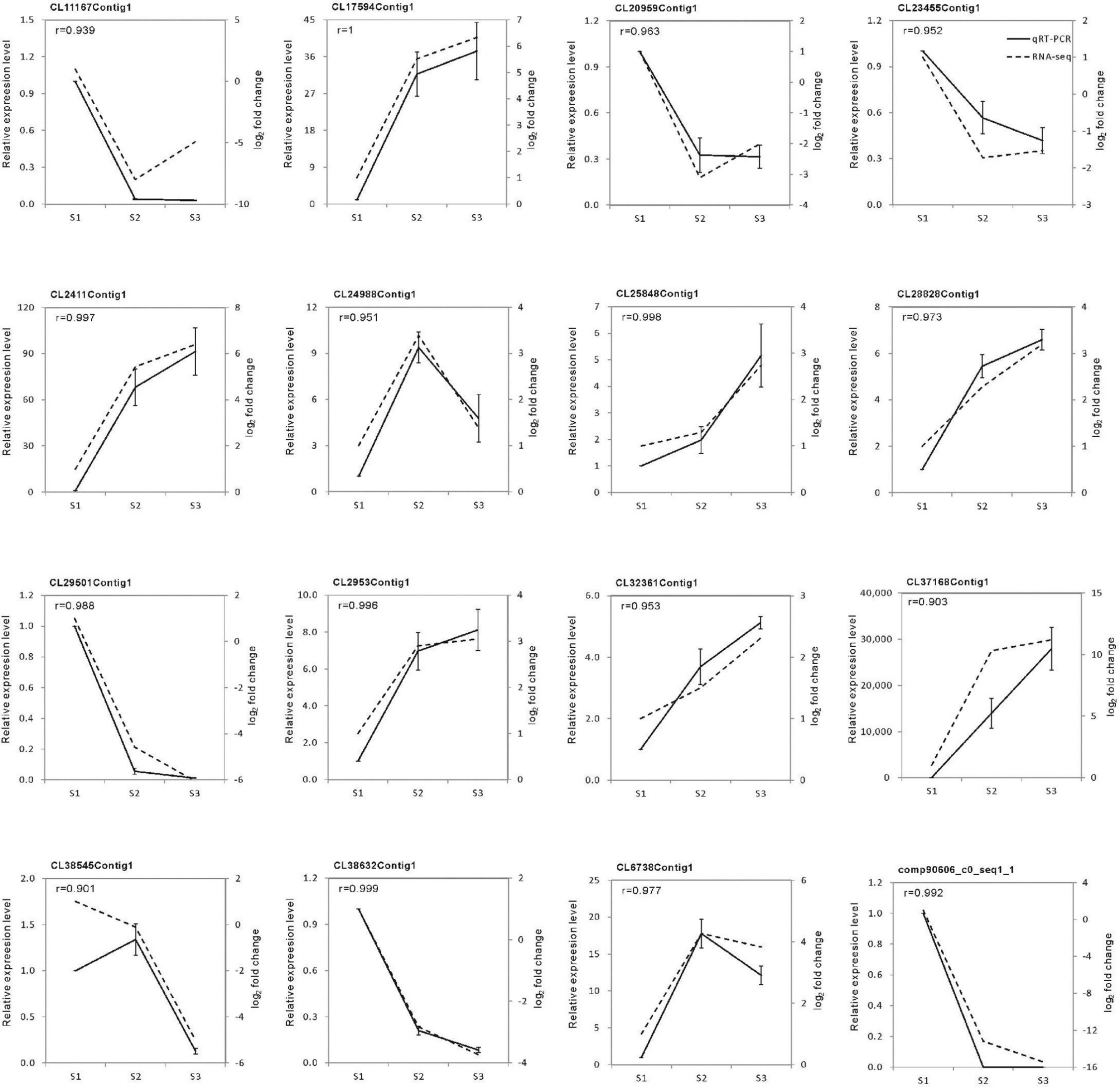
RT-qPCR validation. *EF1a* was used as the internal control. The error bars represent the SE of the RT-qPCR data (*n* = 3). “r” represents the Pearson correlation coefficient. Pearson correlation between the RNA-Seq data and RT-qPCR data was calculated using the log_2_ fold change and the relative expression level.

To confirm the reliability of the RNA-Seq data, the relative expression levels of 16 DEGs were examined by RT-qPCR (Fig 6). PCR amplification indicated that all the primers for RT-qPCR generated only single segments with the expected lengths (61–134 bp, S1 Table). The RT-qPCR results for the randomly selected unigenes were generally consistent with their transcript abundance changes determined by DEG profiling, which confirmed the reliability of the RNA-Seq data. However, discrepancies were observed between the fold change in CL37168Contig1 (in S2) and CL38545Contig1 (in S1) (Fig 6).

## Discussion

The fruit of *L*. *ruthenicum* is renowned for its high anthocyanin content, and is thus valued for its nutritional contribution to human health [4, 33]. The measured color coordinates L*, a*, and b* of fruits indicated that, the lightness and yellow color were declined, and the red color increased definitely with fruit maturation (Fig 1A and 1B). The total anthocyanin content of *L*. *ruthenicum* fruits at the complete ripeness stage under our experimental conditions corroborated previous reports by Shen et al. [36] and Zeng et al. [37]. The anthocyanin content increased 3.4-fold from S1 to S3 (Fig 1C), suggesting that anthocyanin accumulation plays a role in the maturation process, and *L*. *ruthenicum* is suitable for studying anthocyanin biosynthesis during fruit maturation.

Comparative transcriptome analysis by RNA-Seq has been effectively used for investigating the genes involved in anthocyanin biosynthesis in several plants, such as *Vitis vinifera* [27], *Solanum melongena* L. [29], *Litchi chinensis* Sonn. [48], and *Lactuca sativa* [49]. In the present study on *L*. *ruthenicum*, a total of 43,573 unigenes were assembled, of which only 54.44% were identified (S2 Table). The proportion of unigenes annotated is higher than that in *L*. *barbarum* (33.62%) [50]. The reason of some unigenes unannotation might be too short to have a characterized protein domain, whereas others with a known protein domain are highly diverged from other genes in the databases. Additionally, unannotated unigenes might represent specific genes with novel functions, and thus warrant further investigation.

The transcriptional response of *L*. *ruthenicum* fruits among three ripeness stages associated with anthocyanin accumulation provides a framework for evaluating important transcriptional changes and their associated physiological mechanisms during the maturation process. A total of 8,123 DEGs were observed between the S2 and S1 stages, whereas only 1,529 DEGs were detected between the S3 and S2 stages (Fig 3). The decreased number of DEGs suggested that most of the genes governing important physiological changes were expressed at the stage of skin color change. Three clusters comprising 9,917 transcripts with significant differential expression at *P* ≤ 0.05 were detected (Fig 4), and probably represent the core transcriptome of *L*. *ruthenicum* fruit development.

The majority of the genes in the three main clusters represented physiological processes that have been documented to occur during fruit development. For example, many genes (Cluster 1) involved in photosynthesis and carbon fixation in photosynthetic organisms were downregulated from S1 to S3, suggesting the shutdown of photosynthesis, as observed in previous study on grapevine [27]. Conversely, the substantial up-regulation of 57 genes related to plant hormone signal transduction indicated that plant hormone depletion might induce the relative expression of genes associated with fruit maturation in *L*. *ruthenicum*. This speculation was supported in many plants such as pea, the cell division and elongation during pea fruit growth were maintained by the hormonal interaction of GA and auxin [51]. Besides, Gene modulation relating to necroptosis was observed during the maturation phase, with genes exhibiting declined expression in S2 and remaining relatively stable in S3, suggesting a role for necroptosis in the transcriptomic reprograming that accompanies maturation [52, 53].

The anthocyanin biosynthesis pathway has been extensively studied in several plant species, including apple [26], pak choi [17], grape [54], and others. In the present study, 25 DEGs implicated in anthocyanin biosynthesis, including *PAL*, *C4H*, *4CL*, *CHS*, *CHI*, *F3H*, *F3’H*, *F3’5’H*, *DFR*, *ANS*, and *UFGT*, were identified (Table 2). Moreover, 16 structural genes were filtered with FPKM ≥ 5 and fold change > 2 in at least one of the three stages during fruit ripening, most of which were significantly up-regulated. Although five structural genes exhibited declined expressional change patterns, including three *4CL* genes (CL6132Contig1, CL41122Contig1 and comp97802_c0_seq7_2), one *CHI* gene (CL31595Contig1), and one *UFGT* gene (CL11932Contig1). In combination, our results confirmed that most of these structural genes were up-regulatedin response to anthocyanin biosynthesis, which is in accordance with previous studies [8, 37], and the expression patterns of half of the members from 11 type enzymes families were highly correlated with the anthocyanin accumulation pattern, suggesting that these genes might play an extensive and important role in anthocyanin biosynthesis.

Anthocyanin biosynthesis is regulated by several well-studied TFs, including MYB, bHLH, and WD40 [13, 19, 55–57]. Yan et al. [38] annotated 83 MYB family TFs in *L*. *ruthenicum* using RNA-Seq, and the observed expression patterns suggested that some MYB TFs might play a role in the regulation of anthocyanin synthesis during different fruit development periods. In the present study, 62 unigenes encoding *MYB* genes and 192 unigenes encoding *MYB-*related genes were identified. Furthermore, we selected two *MYB* genes and 19 *MYB-*related genes with FPKM ≥ 50 in at least one of the three stages during fruit ripening, and found that the transcripts of the 13 genes decreased significantly during maturation. The results implied that the anthocyanin biosynthetic pathway might be partially negatively controlled by MYB repressors in *L*. *ruthenicum*. Similar phenomena have been observed in many plants, such as *Arabidopsis* [58], strawberry [59], and plum [8]. In addition, an *MYB* gene (CL450Contig1) was significantly positively correlated with five structural genes, including a *CHS* gene (CL25405Contig1, *r* = 1.000**), a *CHI* gene (CL25937Contig1, *r* = 0.998*), an *F3H* gene (CL6713Contig1, *r* = 1.000**), a *DFR* gene (CL6494Contig1, *r* = 1.000**), and an *ANS* gene (CL29803Contig1, *r* = 1.000**) (S3 Table). Moreover, another set of five *MYB* genes were also significantly correlated with at least five structural genes. This finding suggested that these *MYB* genes might interact with structural genes related to anthocyanin biosynthesis. In *Arabidopsis* and rice, 162 and 167 bHLH-encoding genes were respectively identified that potentially participate in a variety of combinatorial interactions, including the capacity to regulate a multitude of transcriptional programs, particularly flavonoid and anthocyanin biosynthesis [22, 23, 60]. We also identified 110 bHLH-encoding genes among the *L*. *ruthenicum* fruit maturation stages, and five genes with FPKM ≥ 50 were selected for further analysis. From this we discovered that four bHLH-encoding genes were evidently up-regulated during fruit ripening, and the orthologues of a bHLH-encoding gene (CL1763Contig1) were identified in *Capsicum annuum* (EU046275.1), *Nicotiana tomentosiformis* (XM_009588892.2) and so on. Interestingly, all the genes were significantly correlated with several structural genes, including *4CL*, *CHS*, *CHI*, *F3’5’H*, and *DFR*, indicating a possible link between bHLH and the structural genes in *L*. *ruthenicum*. Yan et al. [38] speculated that *LrAN11*, encoding a WD40 repeat protein, might be involved in the regulation of anthocyanin biosynthesis in *L*. *ruthenicum*. Our results indicated that CL30472Contig1 encoding *LrAN11* (KY131959) accumulated at higher levels in the later stages of ripening, and showed a significant correlation with structural genes, such as a *4CL* gene (CL32840Contig1, *r* = 1.000**), a *CHI* gene (CL25937Contig1, *r* = 0.998*), and an *F3’5’H* gene (CL6842Contig1, *r* = 1.000**). Further analysis is required to verify whether the identified candidate genes are related to anthocyanin biosynthesis in *L*. *ruthenicum*.

**Fig 7:**
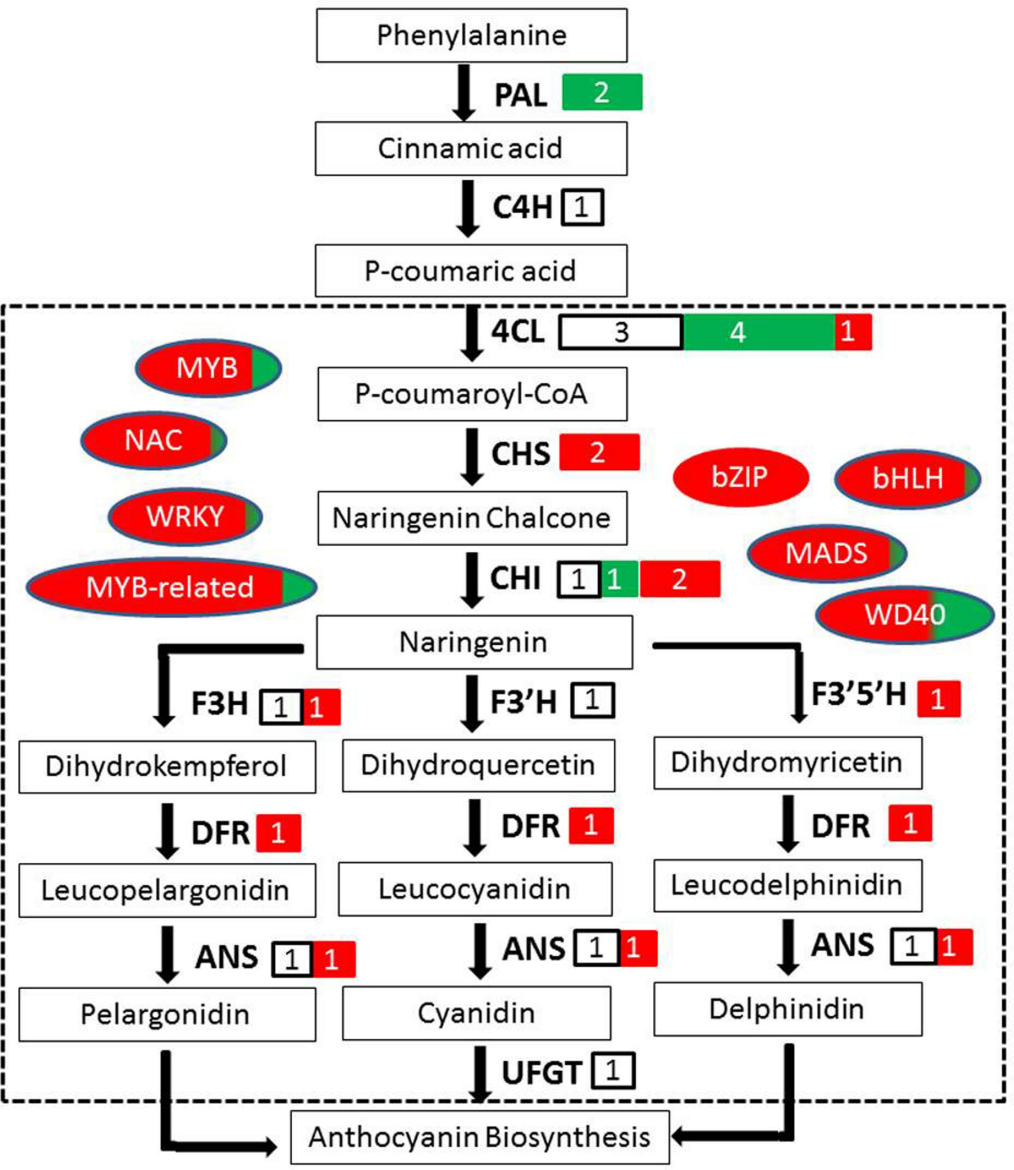
The inferred anthocyanin biosynthesis pathway in *L*. *ruthenicum* fruit. The structural genes including phenylalanine ammonia-lyase (PAL), cinnamate-4-hydroxylase (C4H), 4-coumaroyl: CoA-ligase (4CL), chalcone synthase (CHS), chalcone isomerase (CHI), flavanone 3-hydroxylase (F3H), flavonoid 3'-hydroxylase (F3’H), flavonoid 3'5'-hydroxylase (F3’5’H), dihydroflavonol 4-reductase (DFR), anthocyanidin synthase (ANS), and UDP-glucose: flavonoid 3-O-glucosyltransferase (UFGT) are indicated in bold. The transcription factors including MYB, MYB-related, bHLH, NAC, WRKY, bZIP, MADS, and WD40 are highlighted in ellipse. White, green and red colors are used to indicate the transcript levels for stable, down- regulated, and up-regulated, respectively.

In addition to the TFs mentioned above, TFs such as WRKY, NAC, bZIP, and MADS have also been implicated in anthocyanin biosynthesis in plants [61–64]. In our study, several genes, including *NAC*, *WRKY*, *bZIP*, and *MADS*, were identified (S3 Table). Among them, two *NAC* genes (CL1987Contig1 and CL28914Contig1) and two *WRKY* genes (CL11403Contig1 and CL16938Contig1) were positively correlated with four and five structural genes, respectively. Furthermore, two *bZIP* genes (CL29599Contig1 and CL39763Contig1) and two *MADS* genes (CL6945Contig1 and CL22157Contig1) were upregulated and significantly positively correlated with six structural genes, implying that they might directly modulate the transcription of structural genes. Interestingly, almost all of the putative TFs were assembled to cluster 3 with increasing expression changes, and all of the TFs were highly correlated with the anthocyanin accumulation (Table 3). From these results, we believed that the high expressions of TFs were coupled with the anthocyanin biosynthesis, eventually leading the fruits to display a purple color.Based on the above results, an anthocyanin biosynthesis pathway in *L*. *ruthenicum* fruit was inferred (Fig 7). Briefly, P-coumaric acid is generated following the negative regulation of *PAL* and the stable regulation of *C4H*. Next, *4CL* plays a key role in the generation of P-coumaroyl-CoA. Then, under the regulation of *CHS*, *CHI*, *F3H*, *F3’H*, *F3’5’H*, *DFR*, *ANS* and *UFGT*, anthocyanin is generated. The anthocyanin transcription factors including MYB, MYB-related, bHLH, NAC, WRKY, bZIP, MADS, and WD40 play roles in the regulation of anthocyanin structural genes expression, although the relationship between these TFs and anthocyanin biosynthesis requires further experimental validation.

## Conclusions

In this study, we evaluated the transcriptome of *L*. *ruthenicum* during fruit development and identified a variety of processes associated with different stages of fruit maturation. There were substantial transcriptomic variations between the different ripeness stages that were correlated with differences in anthocyanin accumulation. Out of a total of 43,573 assembled unigenes, 12,734 unigenes were differentially expressed during fruit ripening in *L*. *ruthenicum*. Candidate genes related to anthocyanin biosynthesis, including structural genes and TFs, were identified, but further analysis is required to confirm their role in anthocyanin biosynthesis. This study is the first comprehensive study using transcriptomic techniques to investigate how anthocyanin biosynthesis responds to fruit maturation in *L*. *ruthenicum*. The results in this study paved groundwork for further functional study on anthocyanin-related genes that will ultimately decipher the mechanism underlying persistentlyhigh anthocyanin contents in *L*. *ruthenicum*, and such knowledge can also be useful for the other berries.

## Supporting information

S1 Fig Species distribution of the BLAST search results in the Nr database. The species distribution of the unigene BLAST results against the Nr database with an E-value cutoff of 10^−5^ was analyzed. Different species are indicated by different colors.
(TIF)

S1 Table Primer sequences for RT-qPCR analysis. (DOCX)

S2 Table Summary of sequencing and *de novo* assembly. (DOCX)

S3 Table Correlation analysis of the TFs and structural genes involved in anthocyanin biosynthesis. ‘*’ means significant correlation at *P*-value < 0.05 (2-tailed), and ‘**’ means significant correlation at *P*-value < 0.01 (2-tailed). ‘*’ and ‘**’ were indicated with the red tag. TFs which had significant correlation with five or more structural genes were showed with the yellow tag, and the blue tag indicates the TFs correlated with none of the structural genes.
(XLSX)

S4 Table Summary of DEGs of S1 vs S2. *P* -value <0.05 and fold change > 2. (TXT)

S5 Table Summary of DEGs of S1 vs S3. *P* -value <0.05 and fold change > 2. (TXT)

S6 Table Summary of DEGs of S2 vs S3. *P* -value <0.05 and fold change > 2. (TXT)

## References

[1] Tanaka Y, Sasaki N, Ohmiya A. Biosynthesis of plant pigments: anthocyanins, betalains and carotenoids. Plant J. 2008; 54(4): 733–749. 10.1111/j.1365-313X.2008.03447.x 18476875 10.1111/j.1365-313X.2008.03447.x

[2] Butelli E, Licciardello C, Zhang Y, Liu J, Mackay S, Bailey P, et al Retrotransposons control fruit-specific, cold-dependent accumulation of anthocyanins in blood oranges. Plant Cell. 2012; 24(3): 1242–1255. 10.1105/tpc.111.095232 22427337 10.1105/tpc.111.095232 PMC3336134

[3] Winkel-Shirley B. Flavonoid biosynthesis. A colorful model for genetics, biochemistry, cell biology, and biotechnology. Plant Physiol. 2001; 126(2): 485–493. 10.1104/pp.126.2.485 11402179 10.1104/pp.126.2.485 PMC1540115

[4] Li YH, Zou XB, Shen TT, Shi JY, Zhao JW, Mel H. Determination of geographical origin and anthocyanin content of black goji berry (Lycium ruthenicum Murr.) using near-infrared spectroscopy and chemometrics. Food Anal Methods 2017; 10(4): 1034–1044. 10.1007/s12161-016-0666-4

[5] Zafra-Stone S, Yasmin T, Bagchi M, Chatterjee A, Vinson JA, Bagchi D. Berry anthocyanins as novel antioxidants in human health and disease prevention. Mol Nutr Food Res. 2007; 51(6): 675–683. 10.1002/mnfr.200700002 17533652 10.1002/mnfr.200700002

[6] Li Q, Zhao P, Li J, Zhang C, Wang L, Ren Z. Genome-wide analysis of the WD-repeat protein family in cucumber and Arabidopsis. Mol Genet Genomics. 2014; 289(1): 103–124. 10.1007/s00438-013-0789-x 24292651 10.1007/s00438-013-0789-x

[7] Sakuta M. Diversity in plant red pigments: anthocyanins and and betacyanins. Plant Biotechnol Rep. 2014; 8(1): 37–48. 10.1007/s11816-013-0294-z

[8] Fang ZZ, Zhou DR, Ye XF, Jiang CC, Pan SL. Identification of candidate anthocyanin-related genes by transcriptomic analysis of ‘Furongli’ plum (Prunus salicina Lindl.) during fruit ripening using RNA-seq. Front Plant Sci. 2016; 7(166). 10.3389/fpls.2016.01338 27630660 10.3389/fpls.2016.01338 PMC5005409

[9] Matsui K, Umemura Y, Ohme-Takagi M. AtMYBL2, a protein with a single MYB domain, acts as a negative regulator of anthocyanin biosynthesis in Arabidopsis. Plant J. 2008; 55(6): 954–967. 10.1111/j.1365-313X.2008.03565.x 18532977 10.1111/j.1365-313X.2008.03565.x

[10] Lin-Wang K, Micheletti D, Palmer J, Volz R, Lozano L, Espley R, et al High temperature reduces apple fruit colour via modulation of the anthocyanin regulatory complex. Plant Cell Environ. 2011; 34(7): 1176–1190. 10.1111/j.1365-3040.2011.02316.x 21410713 10.1111/j.1365-3040.2011.02316.x

[11] Jiang M, Ren L, Lian H, Liu Y, Chen H. Novel insight into the mechanism underlying light-controlled anthocyanin accumulation in eggplant (Solanum melongena L.). Plant Sci. 2016; 249: 46–58. 10.1016/j.plantsci.2016.04.001 27297989 10.1016/j.plantsci.2016.04.001

[12] Jin X, Huang H, Wang L, Sun Y, Dai S. Transcriptomics and metabolite analysis reveals the molecular mechanism of anthocyanin biosynthesis branch pathway in different *Senecio cruentus* cultivars. Front Plant Sci. 2016; 7(107): 1307 10.3389/fpls.2016.01307 27656188 10.3389/fpls.2016.01307 PMC5012328

[13] Jin W, Wang H, Li M, Wang J, Yang Y, Zhang X, et al The R2R3 MYB transcription factor PavMYB10.1 involves in anthocyanin biosynthesis and determines fruit skin colour in sweet cherry (Prunus avium L.). Plant Biotechnol J. 2016; 14(11): 2120–2133. 10.1111/pbi.12568 27107393 10.1111/pbi.12568 PMC5095807

[14] Feller A, Machemer K, Braun EL, Grotewold E. Evolutionary and comparative analysis of MYB and bHLH plant transcription factors. Plant J. 2011; 66, 94–116. 10.1111/j.1365-313X.2010.04459.x 21443626 10.1111/j.1365-313X.2010.04459.x

[15] Heppel SC, Jaffé FW, Takos AM, Schellmann S, Rausch T, Walker AR, et al Identification of key amino acids for the evolution of promoter target specificity of anthocyanin and proanthocyanidin regulating MYB factors. Plant Mol Biol. 2013; 82(4–5): 457–471. 10.1007/s11103-013-0074-8 23689818 10.1007/s11103-013-0074-8

[16] Albert NW. Subspecialization of R2R3-MYB repressors for anthocyanin and proanthocyanidin regulation in forage legumes. Front Plant Sci. 2015; 6: 1165 10.3389/fpls.2015.01165 26779194 10.3389/fpls.2015.01165 PMC4689181

[17] Zhang L, Xu B, Wu T, Yang YF, Fan LX, Wen MX, et al Transcriptomic profiling of two Pak Choi varieties with contrasting anthocyanin contents provides an insight into structural and regulatory genes in anthocyanin biosynthetic pathway. Bmc Genomics. 2017; 18(1): 288 10.1186/s12864-017-3677-7 28399809 10.1186/s12864-017-3677-7 PMC5387373

[18] Borevitz JO, Xia Y, Blount J, Dixon RA, Lamb C. Activation tagging identifies a conserved MYB regulator of phenylpropanoid biosynthesis. Plant Cell. 2000; 12: 2383–2393. 10.1105/tpc.12.12.2383 11148285 10.1105/tpc.12.12.2383 PMC102225

[19] Gonzalez A, Zhao M, Leavitt JM, Lloyd AM. Regulation of the anthocyanin biosynthetic pathway by the TTG1/bHLH/Myb transcriptional complex in Arabidopsis seedlings. Plant J. 2008; 53(5): 814–827. 10.1111/j.1365-313X.2007.03373.x 18036197 10.1111/j.1365-313X.2007.03373.x

[20] Paz-Ares J, Ghosal D, Wienand U, Peterson PA, Saedler H. The regulatory *c1* locus of *Zea mays* encodes a protein with homology to myb proto-oncogene products and with structural similarities to transcriptional activators. EMBO J. 1987; 6(12): 3553–3558. 3428265 10.1002/j.1460-2075.1987.tb02684.x PMC553820

[21] Schwinn KE, Venail J, Shang Y, Mackay S, Alm V, Butelli E, et al A small family of MYB-regulatory genes controls floral pigmentation intensity and patterning in the genus Antirrhinum. Plant Cell. 2006; 18: 831–851. 10.1105/tpc.105.039255 16531495 10.1105/tpc.105.039255 PMC1425845

[22] Bailey PC, Martin C, Toledo-Ortiz G, Quail PH, Huq E, Heim MA, et al Update on the basic helix-loop-helix transcription factor gene family in *Arabidopsis thaliana*. Plant Cell. 2003; 15 (11): 2497–2501. 10.1105/tpc.151140 14600211 10.1105/tpc.151140 PMC540267

[23] Li X, Duan X, Jiang H, Sun Y, Tang Y, Yuan Z, et al Genome-wide analysis of basic helix-loophelix transcription factor family in rice and Arabidopsis. Plant Physiol. 2006; 141 (4): 1167–1184. 10.1104/pp.106.080580 16896230 10.1104/pp.106.080580 PMC1533929

[24] Miller JC, Chezem WR, Clay NK. Ternary WD40 repeat-containing protein complexes: evolution, composition and roles in plant immunity. Front Plant Sci. 2016; 6:1108 10.3389/fpls.2015.01108 26779203 10.3389/fpls.2015.01108 PMC4703829

[25] Iaria DL, Chiappetta A, Muzzalupo I. A de novo transcriptomic approach to identify flavonoids and anthocyanins "switch-off" in olive (Olea europaea L.) drupes at different stages of maturation. Front Plant Sci. 2016; 6: 1246 10.3389/fpls.2015.01246 26834761 10.3389/fpls.2015.01246 PMC4717290

[26] Hu DG, Sun CH, Ma QJ, You CX, Cheng L, Hao YJ. MdMYB1 regulates anthocyanin and malate accumulation by directly facilitating their transport into vacuoles in apples. Plant Physiol. 2016; 170(3): 1315–1330. 10.1104/pp.15.01333 26637549 10.1104/pp.15.01333 PMC4775115

[27] Massonnet M, Fasoli M, Tornielli GB, Altieri M, Sandri M, Zuccolotto P, et al Ripening transcriptomic program in red and white grapevine varieties correlates with berry skin anthocyanin accumulation. Plant Physiol. 2017; 174(4): 2376–2396. 10.1104/pp.17.00311 28652263 10.1104/pp.17.00311 PMC5543946

[28] Pantelidis GE, Vasilakakis M, Manganaris GA, Diamantidis G. Antioxidant capacity, phenol, anthocyanin and ascorbic acid contents in raspberries, blackberries, red currants, gooseberries and cornelian cherries. Food Chem. 2007; 102(3): 777–783. 10.1016/j.foodchem.2006.06.021

[29] Li J, Ren L, Gao Z, Jiang M, Liu Y, Zhou L, et al Combined transcriptomic and proteomic analysis constructs a new model for light-induced anthocyanin biosynthesis in eggplant (Solanum melongena L.). Plant Cell Environ. 2017; 40(12): 3069–3087. 10.1111/pce.13074 28940206 10.1111/pce.13074

[30] Hu B, Zhao J, Lai B, Qin Y, Wang H, Hu G. LcGST4 is an anthocyanin-related glutathione S-transferase gene in *Litchi chinensis* Sonn. Plant Cell Rep. 2016; 35(4): 831–843. 10.1007/s00299-015-1924-4 26743425 10.1007/s00299-015-1924-4

[31] Dhar P, Tayade A, Ballabh B, Chaurasia OP, Bhatt RP, Srivastava RB. Lycium ruthenicum Murray: a less-explored but high-value medicinal plant from Trans-Himalayan cold deserts of Ladakh, India. Plant Archives. 2011; 11(2): 583–586.

[32] Peng Q, Xu Q, Yin H, Huang L, Du Y. Characterization of an immunologically active pectin from the fruits of *Lycium ruthenicum*. Int J Biol Macromol. 2014; 64(2): 69–75. 10.1016/j.ijbiomac.2013.11.030 24315943 10.1016/j.ijbiomac.2013.11.030

[33] Zheng J, Ding CX, Wang LS, Li GL, Shi JY, Li H, et al Anthocyanins composition and antioxidant activity of wild *Lycium ruthenicum* Murr. from Qinghai-Tibet Plateau. Food Chem. 2011; 126(3): 859–865. 10.1016/j.foodchem.2010.11.052

[34] Chen HK, Feng Y, Wang LN, Yonezawa T, Crabbe MJ, Zhang X, et al Transcriptome profiling of the UV-B stress response in the desert shrub *Lycium ruthenicum*. Mol Biol Rep. 2015; 42: 639–649. 10.1007/s11033-014-3809-y 25366177 10.1007/s11033-014-3809-y

[35] Chen C, Shao Y, Tao YD, Mei LJ, Shu QY, Wang LS. Main anthocyanins compositions and corresponding H-ORAC assay for wild Lycium ruthenicum Murr. fruits from the Qaidam Basin. J Pharm Tech & Drug Res. 2013; 2(1):1 10.1080/15287394.2018.1451180

[36] Shen XF, Zeng SH, Wu M, Liu CZ, Wang Y. Characterization of proanthocyanin-related leucoanthocyanidin reductase and anthocyanidin reductase genes in Lycium ruthenicum Murr. J Chinese Pharm Sci. 2014; 23(6): 369–377. 10.5246/jcps.2014.06.050

[37] Zeng S, Wu M, Zou C, Liu X, Shen X, Hayward A, et al Comparative analysis of anthocyanin biosynthesis during fruit development in two Lycium species. Physiol Plant. 2014; 150(4): 505–516. 10.1111/ppl.12131 24661321 10.1111/ppl.12131

[38] Yan L, Wang CP, Chen JW, Qiao GX, Li J. Analysis of MYB transcription factor family based on transcriptome sequencing in Lycium ruthenicum Murr. Scientia Agricultura Sinica. 2017; 50(20): 3991–4002. 10.3864/j.issn.0578-1752.2017.20.013

[39] Wang Y, Ding L, Wang SQ. Study on proanthocyanidins and anthocyanins contents of Lycium ruthenicum Murr. from different areas (in Chinese). Science and Technology of Food Industry. 2016, 37(13): 122–126. doi: 10.13386/j.issn1002-0306

[40] Cui Y, Jiang CY, Zhang X, He HY, Tao XL, Wang R. Extraction and Content Analysis of Anthocyanin from Lycium Ruthenicum Murr. (in Chinese). 2017; 38(16): 28–32. 10.3969/j.issn.1005-6521.2017.16.007

[41] Yan YM, Dai GL, Ran LW, Luo Q, Li XY, Qin K, et al The polyphenols composition of Lycium ruthenicum Murr. from different places (in Chinese). 2014; 47(22): 4540–4550. 10.3864/j.issn.0578-1752.2014.22.020

[42] Schroeder A, Mueller O, Stocker S, Salowsky R, Leiber M, Gassmann M, et al The RIN: an RNA integrity number for assigning integrity values to RNA measurements. BMC Mol Biol. 2006; 7: 3 10.1186/1471-2199-7-3 16448564 10.1186/1471-2199-7-3 PMC1413964

[43] Patel RK, Jain M. NGS QC Toolkit: a toolkit for quality control of next generation sequencing data. PLoS ONE. 2012; 7(2): p. e30619 10.1371/journal.pone.0030619 22312429 10.1371/journal.pone.0030619 PMC3270013

[44] Dang ZH, Zheng LL, Wang J, Gao Z, Wu SB, Qi Z, et al Transcriptomic profiling of the salt-stress response in the wild recretohalophyte *Reaumuria trigyna*. BMC Genomics. 2013; 14: 29 10.1186/1471-2164-14-29 23324106 10.1186/1471-2164-14-29 PMC3562145

[45] Li CY, Liu XM, Qiang XN, Li XY, Li XS, Zhu SR, et al EBP1 nuclear accumulation negatively feeds back on FERONIA-mediated RALF1 signaling. PLoS Biol. 2018; 16(10): e2006340 10.1371/journal.pbio.2006340 30339663 10.1371/journal.pbio.2006340 PMC6195255

[46] Ernst J, Nau GJ, Bar-joseph Z. Clustering short time series gene expression data. Bioinformatics. 2005; 21 Suppl 1: i159–i168. 10.1093/bioinformatics/bti1022 15961453 10.1093/bioinformatics/bti1022

[47] Livak KJ, Schmittgen TD. Analysis of relative gene expression data using real-time quantitative PCR and the 2^−ΔΔCT^ method. Methods. 2001; 25(4): 402–408. 10.1006/meth.2001.1262 11846609 10.1006/meth.2001.1262

[48] Zhang HN, Li WC, Wang HC, Shi SY, Shu B, Liu LQ, et al Transcriptome profiling of light-regulated anthocyanin biosynthesis in the pericarp of litchi. Front Plant Sci. 2016; 7(225): 963 10.3389/fpls.2016.00963 27446187 10.3389/fpls.2016.00963 PMC4925703

[49] Zhang YZ, Xu SZ, Cheng YW, Ya HY, Han JM. Transcriptome analysis and anthocyanin-related genes in red leaf lettuce. Genet Mol Res. 2016; 15(1). 10.4238/gmr.15017023 26909931 10.4238/gmr.15017023

[50] Chen CC, Xu ML, Wang CP, Qiao GX, Wang WW, Tan ZY, et al Characterization of the Lycium barbarum fruit transcriptome and development of EST-SSR markers. 2017 PLoS ONE, 12(11): e0187738 10.1371/journal.pone.0187738 29125846 10.1371/journal.pone.0187738 PMC5695279

[51] Ozga JA, Huizen RV, Reinecke DM. Hormone and seed-specific regulation of pea fruit growth. Plant Physiol. 2002; 128(4):1379–89. 10.1104/pp.010800 11950986 10.1104/pp.010800 PMC154265

[52] Lin L, Zakeri ZF. Apoptosis in development. Methods Mol Biol. 2000; 407(6805): 796–801. 10.1385/1-59259-065-9:107

[53] Ameisen JC. On the origin, evolution, and nature of programmed cell death: a timeline of, four billion years. Cell Death Differ. 2002; 9(4): 367–393. 10.1038/sj/cdd/4400950 11965491 10.1038/sj.cdd.4400950

[54] Matus JT, Cavallini E, Loyola R, Höll J, Finezzo L, Dal Santo S, et al A group of grapevine MYBA transcription factors located in chromosome 14 control anthocyanin synthesis in vegetative organs with different specificities compared with the berry color locus. Plant J. 2017; 91(2): 220–236. 10.1111/tpj.13558 28370629 10.1111/tpj.13558

[55] Ramsay NA, Glover BJ. MYB-bHLH-WD40 protein complex and the evolution of cellular diversity. Trends Plant Sci. 2005; 10(2): 63–70. 10.1016/j.tplants.2004.12.011 15708343 10.1016/j.tplants.2004.12.011

[56] Li P, Chen B, Zhang G, Chen L, Dong Q, Wen J, et al Regulation of anthocyanin and proanthocyanidin biosynthesis by *Medicago truncatula* bHLH transcription factor MtTT8. New Phytol. 2016; 210(3): 905–921. 10.1111/nph.13816 26725247 10.1111/nph.13816

[57] Cao X, Qiu Z, Wang X, Van Giang T, Liu X, Wang J. et al A putative R3 MYB repressor is the candidate gene underlying atroviolacium, a locus for anthocyanin pigmentation in tomato fruit. J Exp Bot. 2017; 68(21–22): 5745–5758. 10.1093/jxb/erx382 29186488 10.1093/jxb/erx382 PMC5854135

[58] Dubos C, Le Gourrierec J, Baudry A, Huep G, Lanet E, Debeaujon I, et al MYBL2 is a new regulator of flavonoid biosynthesis in *Arabidopsis thaliana*. Plant J. 2008; 55(6): 940–953. 10.1111/j.1365-313X.2008.03564.x 18532978 10.1111/j.1365-313X.2008.03564.x

[59] Salvatierra A, Pimentel P, Moya-Leon MA, Herrera R. Increased accumulation of anthocyanins in *Fragaria chiloensis* fruits by transient suppression of *FcMYB1* gene. Phytochemistry. 2013; 90: 25–36. 10.1016/j.phytochem.2013.02.016 23522932 10.1016/j.phytochem.2013.02.016

[60] Toledo-Ortiz G, Huq E, Quail PH. The Arabidopsis basic/helixloop-helix transcription factor family. Plant Cell. 2003; 15(8): 1749–1770. 10.1105/tpc.013839 12897250 10.1105/tpc.013839 PMC167167

[61] Jaakola L. New insights into the regulation of anthocyanin biosynthesis in fruits. Trends Plant Sci. 2013; 18(9): 477–483. 10.1016/j.tplants.2013.06.003 23870661 10.1016/j.tplants.2013.06.003

[62] Xu W, Grain D, Bobet S, Le Gourrierec J, Thevenin J, Kelemen Z, et al Complexity and robustness of the flavonoid transcriptional regulatory network revealed by comprehensive analyses of MYB-bHLH-WDR complexes and their targets in Arabidopsis seed. New Phytol. 2014; 202(1): 132–144. 10.1111/nph.12620 24299194 10.1111/nph.12620

[63] Zhou H, Lin-Wang K, Wang H, Gu C, Dare AP, Espley RV, et al Molecular genetics of blood-fleshed peach reveals activation of anthocyanin biosynthesis by NAC transcription factors. Plant J. 2015; 82(1): 105–121. 10.1111/tpj.12792 25688923 10.1111/tpj.12792

[64] Chen F, Hu Y, Vannozzi A, Wu K, Cai HY, Qin Y, et al The WRKY transcription factor family in model plants and crops. Crit Rev Plant Sci. 2018; 1–25. 10.1080/07352689.2018.1441103

