# Supplementary figures and images for: Transcriptomic analysis of Lycium ruthenicum Murr. during fruit ripening provides insight into structural and regulatory genes in the anthocyanin biosynthetic pathway
Source: PLoS One. 2018 Dec 7;13(12):e0208627. doi: 10.1371/journal.pone.0208627 (PMC6285980; doi:10.1371/journal.pone.0208627)

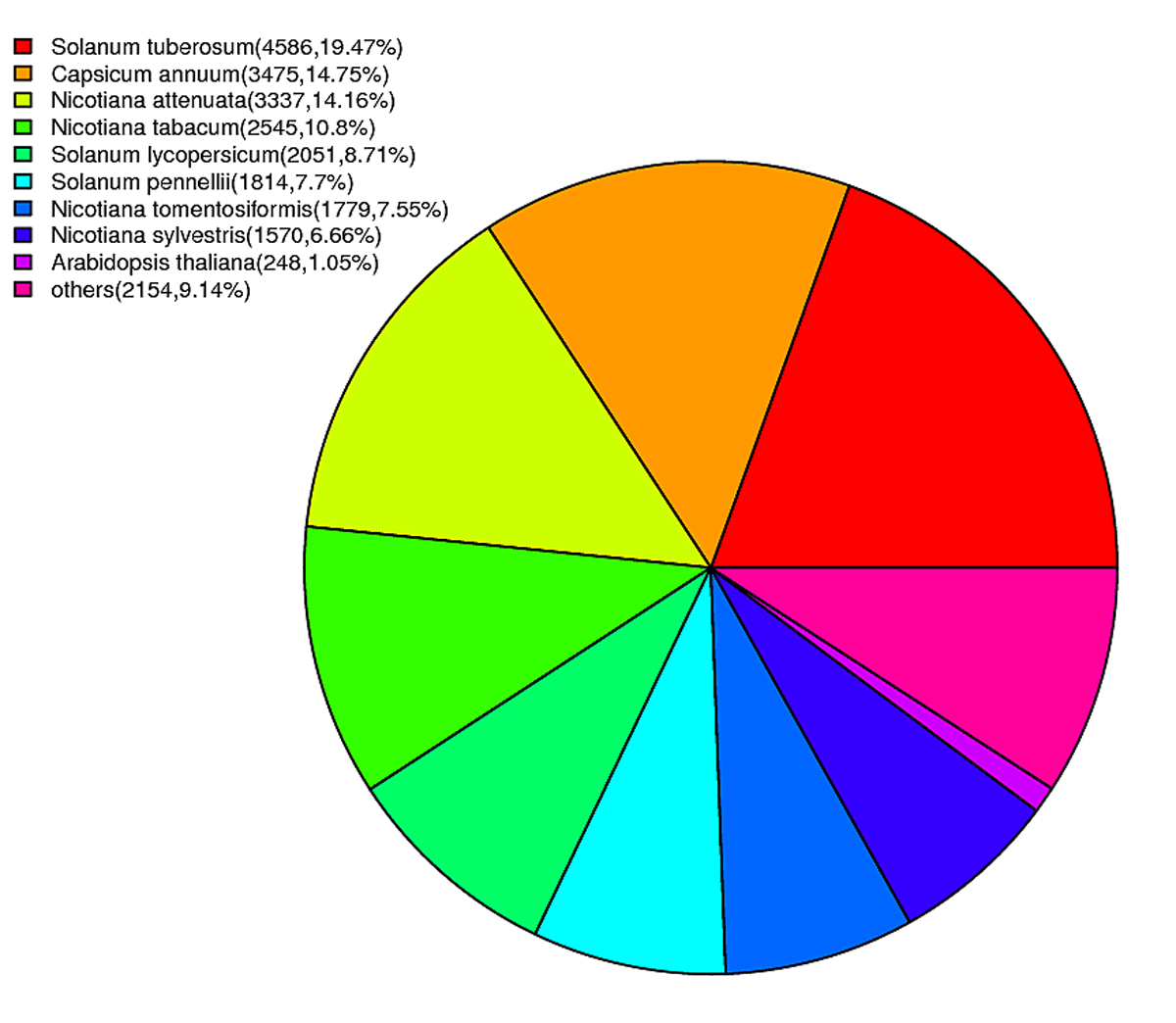

Supplement: S1 Fig — The species distribution of the unigene BLAST results against the Nr database with an E-value cutoff of 10−5 was analyzed. Different species are indicated by different colors. (TIF) [file pone.0208627.s001.tif]
