# Supplementary material for: Transcriptomic analysis of Lycium ruthenicum Murr. during fruit ripening provides insight into structural and regulatory genes in the anthocyanin biosynthetic pathway
Source: PLoS One. 2018 Dec 7;13(12):e0208627. doi: 10.1371/journal.pone.0208627 (PMC6285980; doi:10.1371/journal.pone.0208627)
Supplement: S1 Table — (DOCX) [file pone.0208627.s002.docx]

**SUPPLEMENTARY TABLE S1 |** Primer sequences for RT-qPCR analysis.

| **Unigene ID** | **Forward primer** | **Reverse primer** | **Product length (bp)** | **Ta (℃)** |
| --- | --- | --- | --- | --- |
| CL11167Contig1 | AGGTCTAGTCCAGTAATGAGC | CCTTTGACAAAGCCTGAATC | 61 | 60 |
| CL17594Contig1 | ACTGTGGATGGAATTCGG | GGCCAACTAATAATAGGCACC | 61 | 60 |
| CL20959Contig1 | AACGTCATTGTTGTTTCTGTAG | CTTCGTAAATGGTTGGCAC | 67 | 60 |
| CL23455Contig1 | AGTACAGACTTGCAGACGAA | CCGGTCGGTGGTCCTAAA | 103 | 60 |
| CL2411Contig1 | GTGCTGAGGAAATTGAACCTAT | ACACCAAACAGGAAGTTTAGTC | 68 | 60 |
| CL24988Contig1 | CTTCCTTCTGCGTGCTAGTTA | CAACGTCAATTCGGTAGGTG | 134 | 60 |
| CL25848Contig1 | CGGATATGTCCAAGCTCTC | GGGTGCAACCTTAGTGATTC | 61 | 60 |
| CL28828Contig1 | GTTGAAGCAATCCAAACTGTG | GCCTTTGTAAATCGTCCGAG | 103 | 60 |
| CL29501Contig1 | TGCTTCCTAAACCAGGGTAT | AACCTCAAGATTACTACACGC | 62 | 60 |
| CL2953Contig1 | TGACGGGTAAGCAATACTCC | GACTTTCTGCATCACCGTAG | 87 | 60 |
| CL29781Contig1 | ATGGTTAAGGCTGGCTTT | AGGACGACCAACTATACTAGG | 69 | 60 |
| CL32361Contig1 | CAGAACACAATATCAGCATCAC | GGCCTAACAACCCATACGAA | 89 | 60 |
| CL37168Contig1 | GCACTAACATTTGCGACG | GCGATGATTTCTTCATCCTGAG | 71 | 60 |
| CL38545Contig1 | ACAATTCACAACCCATCTCTT | ACGACTATTTGCCAATCCC | 80 | 60 |
| CL38632Contig1 | TGCGGTCATTGCTAGTCTTA | AGAGCCCTTTAGTCCTACG | 115 | 60 |
| CL6738Contig1 | AGGCTAATCGCATCACAG | CATTACCCAAATAAATGGTCGT | 75 | 60 |
| comp90606_c0_seq1_1 | CAAAGGCGAGGTTGGAAT | CTATCTCTAGCTGCAAATGCG | 76 | 60 |
| EF1a (JX427553) | CCATACCAGCATCACCATTCTTC | GTCACACTTCCCACATTGCC | 117 | 60 |
