# Supplementary material for: Transcriptomic analysis of Lycium ruthenicum Murr. during fruit ripening provides insight into structural and regulatory genes in the anthocyanin biosynthetic pathway
Source: PLoS One. 2018 Dec 7;13(12):e0208627. doi: 10.1371/journal.pone.0208627 (PMC6285980; doi:10.1371/journal.pone.0208627)
Supplement: S2 Table — (DOCX) [file pone.0208627.s003.docx]

**SUPPLEMENTARY TABLE S1 |** Summary of sequencing and *de novo* assembly.

| **Database** | **Annotation numbers** | **Annotation ratio (%)** |
| --- | --- | --- |
| NR | 23559 | 54.07 |
| SWISSPROT | 17212 | 39.50 |
| KOG | 13128 | 30.13 |
| KEGG | 4951 | 11.36 |
| GO | 15064 | 34.57 |
| ALL | 23723 | 54.44 |
